# The MOMENTUM Study: An International Registry for the Evidence-Based Introduction of MR-Guided Adaptive Therapy

**DOI:** 10.3389/fonc.2020.01328

**Published:** 2020-09-07

**Authors:** Sophie R. de Mol van Otterloo, John P. Christodouleas, Erwin L. A. Blezer, Hafid Akhiat, Kevin Brown, Ananya Choudhury, Dave Eggert, Beth A. Erickson, Corinne Faivre-Finn, Clifton D. Fuller, Joel Goldwein, Shaista Hafeez, Emma Hall, Kevin J. Harrington, Uulke A. van der Heide, Robert A. Huddart, Martijn P. W. Intven, Anna M. Kirby, Susan Lalondrelle, Claire McCann, Bruce D. Minsky, Stella Mook, Marlies E. Nowee, Uwe Oelfke, Kristina Orrling, Arjun Sahgal, Jeffrey G. Sarmiento, Christopher J. Schultz, Robbert J. H. A. Tersteeg, Rob H. N. Tijssen, Alison C. Tree, Baukelien van Triest, William A. Hall, Helena M. Verkooijen

**Affiliations:** ^1^Department of Radiation Oncology, University Medical Center Utrecht, Utrecht, Netherlands; ^2^Elekta AB, Stockholm, Sweden; ^3^Division of Imaging, University Medical Center Utrecht, Utrecht, Netherlands; ^4^The Christie National Health Service Foundation Trust, Manchester, United Kingdom; ^5^Department of Radiation Oncology, Medical College of Wisconsin, Milwaukee, WI, United States; ^6^Department of Radiation Oncology, University of Texas MD Anderson Cancer Center, Houston, TX, United States; ^7^The Royal Marsden NHS Foundation Trust and the Institute of Cancer Research, London, United Kingdom; ^8^Clinical Trials and Statistics Unit, The Institute of Cancer Research, London, United Kingdom; ^9^Department of Radiation Oncology, Netherlands Cancer Institute, Antoni van Leeuwenhoek Hospital, Amsterdam, Netherlands; ^10^Department of Radiation Oncology, Sunnybrook Health Sciences Center/Odette Cancer Center, Toronto, ON, Canada; ^11^Lygature, Utrecht, Netherlands

**Keywords:** MR-linac, MRI, functional imaging, radiotherapy, magnetic resonance imaging, image-guidance, adaptive radiotherapy, MR-guided radiation therapy (MRgRT)

## Abstract

**Purpose:** MR-guided Radiation Therapy (MRgRT) allows for high-precision radiotherapy under real-time MR visualization. This enables margin reduction and subsequent dose escalation which may lead to higher tumor control and less toxicity. The Unity MR-linac (Elekta AB, Stockholm, Sweden) integrates a linear accelerator with a 1.5T diagnostic quality MRI and an online adaptive workflow. A prospective international registry was established to facilitate the evidence-based implementation of the Unity MR-linac into clinical practice, to systemically evaluate long-term outcomes, and to aid further technical development of MR-linac-based MRgRT.

**Methods and Results:** In February 2019, the Multi-OutcoMe EvaluatioN of radiation Therapy Using the MR-linac study (MOMENTUM) started within the MR-linac Consortium. The MOMENTUM study is an international academic-industrial partnership between several hospitals and industry partner Elekta. All patients treated on the MR-linac are eligible for inclusion in MOMENTUM. For participants, we collect clinical patient data (e.g., patient, tumor, and treatment characteristics) and technical patient data which is defined as information generated on the MR-linac during treatment. The data are captured, pseudonymized, and stored in an international registry at set time intervals up to two years after treatment. Patients can choose to provide patient-reported outcomes and consent to additional MRI scans acquired on the MR-linac. This registry will serve as a data platform that supports multicenter research investigating the MR-linac. Rules and regulations on data sharing, data access, and intellectual property rights are summarized in an academic-industrial collaboration agreement. Data access rules ensure secure data handling and research integrity for investigators and institutions. Separate data access rules exist for academic and industry partners. This study is registered at ClinicalTrials.gov with ID: NCT04075305 (https://clinicaltrials.gov/ct2/show/NCT04075305).

**Conclusion:** The multi-institutional MOMENTUM study has been set up to collect clinical and technical patient data to advance technical development, and facilitate evidenced-based implementation of MR-linac technology with the ultimate purpose to improve tumor control, survival, and quality of life of patients with cancer.

## Background and Rationale

Radiotherapy is an important pillar in the multimodality treatment of cancer. Recently, MR-guided Radiation Therapy (MRgRT) has been introduced, enabling high-precision radiotherapy under real-time MRI visualization (1, 2). Real-time visualization during MR-guided radiotherapy holds promise for margin reduction and dose escalation, which may lead to higher cure rates and less toxicity (3–5). The Unity MR-linac (Elekta AB, Stockholm, Sweden), integrating a 7 MV linear accelerator (linac) with a 1.5T diagnostic MRI scanner and an online adaptive workflow, enables MRgRT (6–10). The Elekta MR-linac received CE marking in June 2018, followed by FDA approval in December 2018 and Health Canada approval in March 2019, permitting commercial release and clinical implementation of this innovative device.

Technical innovations in radiation oncology, such as the MR-linac, are typically received with great enthusiasm by radiation oncologists and physicists, who are keen to see new technologies implemented in routine practice. Evidence supporting these new radiotherapy technologies is generally scarce (11). However, it is important to evaluate these novel and often costly technologies to gain insight into whether theoretical advantages are translated into actual patient benefits (12). In 2017, Verkooijen et al. (13) introduced the R-IDEAL framework as an assessment methodology for evidence-based clinical evaluation of innovations in radiation oncology. The R-IDEAL model, which was adapted from the surgical IDEAL framework, describes the clinical development process in six stages (14). The process starts with radiotherapy predicate studies (stage 0), followed by first time use of the technology (stage 1: idea), technical optimization (stage 2a: development), proof of early clinical effectiveness, and safety (stage 2b: exploration) and comparison of the innovation against standard care (stage 3: assessment). The final stage, stage 4: long-term evaluation, is crucial for post-marketing and surveillance purposes and evaluates long-term outcomes.

In line with the R-IDEAL framework, The Multi-OutcoMe EvaluatioN of radiation Therapy Using the MR-linac Study (The MOMENTUM study) was established. The goals of the MOMENTUM study are to aid and accelerate the development of anatomic and functional MRgRT and to enable systematic evaluation of clinical outcomes of patients. Ultimately, the MOMENTUM study aims to assess the effectiveness and safety of MRgRT (R-IDEAL stage 2a and 2b) as the paradigm is extended beyond conventional approaches, thereby facilitating the evidence-based introduction of the MR-linac into clinical practice. In addition, this registry is designed to serve as a data platform for future research investigating the MR-linac.

In this article, we describe the MOMENTUM study, a clinical and technical patient registry, the governance structure and the handling of patient confidentiality.

## Methods and Results

### Aims of the MOMENTUM Study

The MOMENTUM study is a complex registry, integrating clinical and technical patient data. The aim of the MOMENTUM study is to provide a data-infrastructure to:

- Collect routine-care data for the evaluation of short- and long-term feasibility, safety, effectiveness, and toxicity of treatments on the MR-linac. This also facilitates evaluation of (early) cost effectiveness of MR-linac treatments.
- Aggregate technical patient data to further develop MR-linac software algorithms that drive the online adaptive workflow aiming to maximize the benefits of MRgRT.
- Create a repository of anatomical and functional MR imaging data supporting Stage 0 of the R-IDEAL framework and aiming to further develop MRgRT.

### The MR-Linac Consortium

The MOMENTUM study was set up within the context of the international MR-linac Consortium, which currently consists of over 30 international centers (15). Four European institutes, two institutes in the United States, one in Canada, and the manufacturer of the MR-linac (Elekta AB, Sweden) were involved in founding the MOMENTUM study (Table 1). All participating Institutes' Committees for the Protection of Human Subjects have approved the MOMENTUM study, and it has been registered at clinicaltrials.gov (https://clinicaltrials.gov/ct2/show/NCT04075305). The MOMENTUM study was aided by a professional public-private partnership manager (Lygature).

**Table 1:** Academic and industry partners of the MOMENTUM study.

**MOMENTUM partners**
University Medical Center Utrecht, NL
The Netherlands Cancer Institute, Antoni van Leeuwenhoek Hospital, NL
Sunnybrook Hospital, CA
MD Anderson Cancer Center, US
Froedtert and Medical College of Wisconsin, US
The Royal Marsden NHS Foundation Trust and The Institute of Cancer Research, UK
The Christie Hospital National Health Service Foundation Trust, UK
Elekta AB, SE

**Figure 1:**
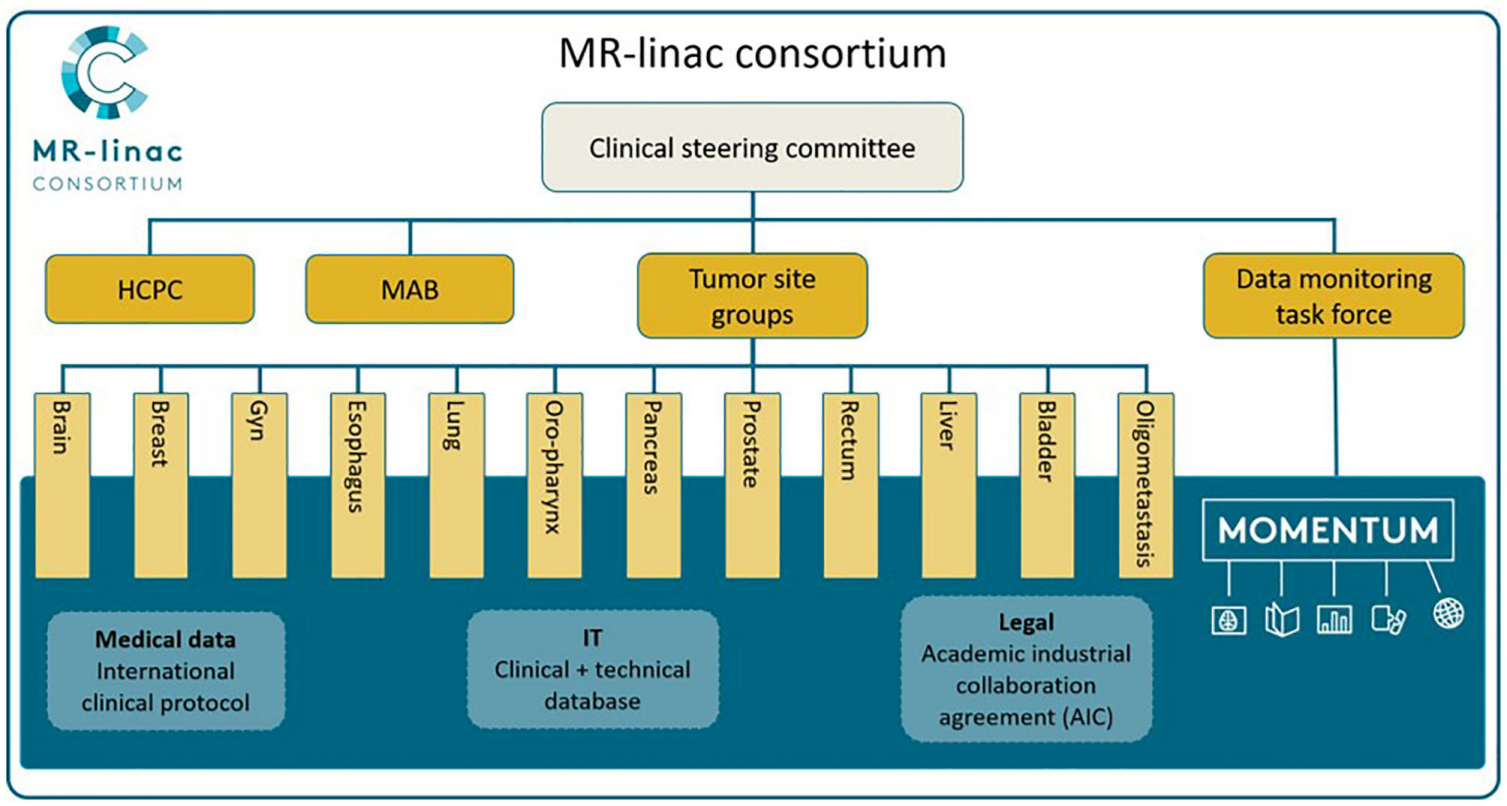
The MR-linac Consortium and its organizational structure. Adapted from “The MRI-Linear Accelerator Consortium: Evidence-Based Clinical Introduction of an Innovation in Radiation Oncology Connecting Researchers, Methodology, Data Collection, Quality Assurance, and Technical Development” by Kerkmeijer et al. (15), Frontiers in Oncology, 6, P. 1–6. HCPC, Health Care Policy Committee; MAB, Methodology Advisory Board.

Twelve Tumor Site Groups (TSGs) were established within the Consortium: brain, bladder, breast, cervix, esophagus, liver, lung, oligometastases, oropharynx, pancreas, prostate, and rectal cancer, as seen in Figure 1 [adapted from Kerkmeijer et al. (15)]. The TSGs are international, cancer site-specific expert panels aiming to develop and evaluate MR-guided treatment strategies. Main activities of these TSGs include designing preparatory studies and collaborative clinical trials that follow the R-IDEAL framework (13). As the MOMENTUM study is an ever evolving and expanding project, both the number of participating centers as the number of TSGs are likely to increase.

The MR-linac Consortium includes a Data Management Task Force (DMTF) which provides oversight and governance and manages the exchange of data according to the data access rules. The DMTF includes radiation oncologists, an Elekta representative, a physicist, and an epidemiologist.

### The MOMENTUM Study's Data Infrastructure

**Figure 2:**
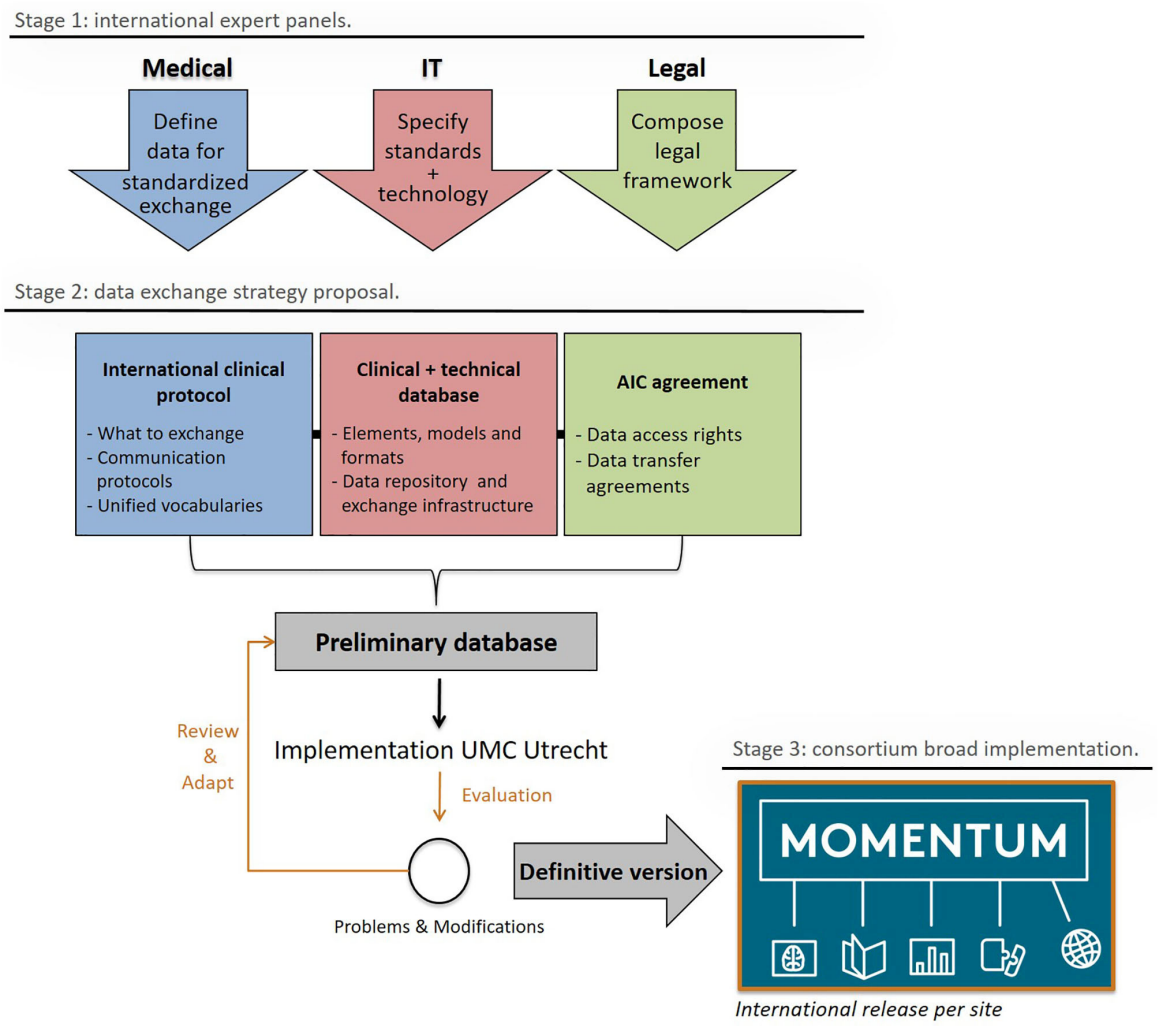
The MOMENTUM exchange strategy and its stages of development: design (1), construction, testing, and amendment (2), and release (3). Adapted from “Creating a data exchange strategy for radiotherapy research: Toward federated databases and anonymized public datasets,” by Skripcak et al. (16), Radiotherapy and Oncology, 113(3), P. 303–309. AIC agreement, Academic Industrial collaboration agreement; IT, Information Technologies.

Figure 2, adapted from Skripcak et al. (16), shows the working scheme for the creation of the MOMENTUM data infrastructure. The MOMENTUM study was created by an international expert panel that addressed the medical, information technological (IT), and legal aspects of the international data infrastructure separately before integrating them into one registry. The MOMENTUM study follows the FAIR criteria (e.g., data being findable, accessible, interoperable, and reproducible) proposed by the Force 11 Group for databases to reach their full potential (17, 18).

#### I. Medical Component

##### Clinical data

For each patient in the MOMENTUM study, a core set of clinical data items is collected, consisting of patient and tumor characteristics and outcome data. These outcome data include toxicity and cancer status data such as recurrence, disease-free, and overall survival (Table 2). Within the MOMENTUM study, patients have the option to provide patient-reported outcomes (PROs) through validated generic and disease-specific quality of life questionnaires. Additionally, the registry was customized to the requirements of each TSG by including TSG-selected and TSG-specific data items. These clinical data items (the core set, the TSG-specific, and PRO data) are collected before, and 3, 6, 12, and 24 months after treatment on the MR-linac. The clinical data are collected in parallel to routine clinical patient follow-up according to local standards of care.

**Table 2:** Detailed data specifications of the clinical and technical patient data collected for the MOMENTUM study.

	**Information type**		**Example**	**Source**	**Time of accrual**	**Data elements and models**	**Terminology & ontology**
**OpenClinica**	Classifier data	Patient	Age, gender, comorbidities	HIS	Baseline	SDTM, CDASH, CDISC	TNM-O, ISO 8601, EQ5D, NCI-Thesaurus, CTCAE, TG263-nomenclature, NAACCR
		Disease	Stage, histology, biomarkers	HIS	Baseline		
		Treatment	Chemotherapy, surgical intent, total RT dose	HIS	End of treatment		
	Outcome data	Toxicity	CTCAE	HIS	3, 6, 12, 24 mos. post-RT		
		Cancer control and PROs	Local control, survival, QoL questionnaires	HIS + patient	3, 6, 12, 24 mos. post-RT		
**Microsoft Azure**	Diagnostic imaging data		Diagnostic CT, MR, PET imaging	PACS	End of treatment	DICOM-RT	Not applicable
	Radiotherapy planning data		Delineation/structure stets, planning CT	PACS, RIS	End of treatment		
	Radiotherapy delivery data		Motion data files, machine log files, fractions	PACS, RIS	End of treatment		

*CDASH, clinical data acquisition standards harmonization; CDISC, clinical data interchange standards consortium; CRC, clinical research coordinator; CT, computerized tomography; CTCAE, common terminology criteria for adverse events; DICOM-RT, digital imaging and communications in medicine in radiation therapy; EQ-5D, standardized instrument for measuring generic health status; HIS, hospital information system; ISO 8601, data elements and interchange formats—information interchange—representation of dates an date and time; Mos, months; NAACR, North American association of central cancer registries; NCI thesaurus, National Cancer Institute's reference terminology and ontology; PACS, picture archiving and communication system; PET, positron emission tomography; PROs, patient reported outcomes; QoL questionnaires, quality of life questionnaires; RIS, radiology information system; RT, radiation therapy; SDTM, study data tabulation model; TG263 nomenclature, standardized nomenclature for radiation therapy; TNM-O, Ontology support for staging of malignant tumors*.

##### Technical patient data

Fundamental to any radiation therapy database is the collection of technical patient data such as imaging information, radiotherapy structures (contours), plans, and dose information. The MOMENTUM study captures these data elements as well as others uniquely offered as a result of the novel, real-time MR-guided adaptive paradigm available on the MR-linac. Technical patient data, ultimately defined as data generated by the MR-linac during clinical operation, are captured after administration of radiotherapy on the MR-linac. Furthermore, researchers can collect extra research MRI scans during the treatment on the MR-linac of patients who consent to the optional research scans.

##### Clinical and technical patient data collection

At every institution, dedicated clinical research coordinators collect clinical and technical patient data from the hospital information system. The information is pseudonymized on site before the coordinators upload the data into the MOMENTUM database. Each patient is assigned a unique study ID (study identification number) which facilitates the link between the technical and clinical patient data. Furthermore, this study ID is used in any future studies on the MR-linac, if applicable.

The study ID allocation is treatment-based, therefore the study ID relates to a radiation treatment course for individual patients. Multiple courses for a single patient are entered as separate events in the database but the IDs are linked facilitating the identification of a single patient with multiple treatment courses.

#### II. IT framework

##### Data pooling architecture

The MOMENTUM clinical registry follows a centralized approach enabling secure virtual storage and easy uploading of data (16). The web-based clinical database (OpenClinica LLC, Waltham MA) is managed and governed by a senior data manager and hosted on a physical server from a neutral zone by the University Medical Center (UMC) Utrecht.

The technical database is hosted on a cloud computing platform (Microsoft Azure, Microsoft Corporation, Redmond, WA) and facilitates uploading of technical patient data, or DICOM data (Digital Imaging and Communications in Medicine) from all over the world. At every site, DICOM data are pseudonymized with the RSNA CTP (Radiological Society of North America Clinical Trial Processor) by a dedicated technical patient data custodian on an on-premise workstation. After pseudonymization, technical patient data are uploaded to the cloud via a secure transfer using Microsoft Fast Data Transfer (Microsoft Corporation, Redmond, WA). Stored data are encrypted and accessible by researchers via secure VPN connection or virtual machines hosted on the cloud.

The clinical and technical repositories function as two separate entities for which the data are only connected through the study ID.

##### Data interoperability

Within the MOMENTUM study, we aim for maximum clinical and technical patient data interoperability according to the previously mentioned FAIR principles (18). The linked data are findable for the MOMENTUM partners within the two comprehensive databases. Straightforward data access by researchers is realized through an online data access request form. Researchers can request specific (multi-institutional) data by submitting this request form directly to the DMTF. Standard data elements defined by the DMTF and standardization of ontology, e.g., by implementing CDISC (Clinical Data Interchange Standards Consortium) standards, TNM-O (Tumor-Node-Metastasis ontology) (19), and ISO 860 (Data elements and interchange formats) are the foundation of the internationally integrated standard of information models effectuating data interoperability within the MOMENTUM study. The MOMENTUM study captures a considerable amount of long-term data. These data can be used in future MR-linac studies which facilitates re-usage of data. Furthermore, we aim to aid in formation of standardized international research protocols for future (non-) intervention studies performed on the MR-linac by serving as a data platform for these studies. This will minimize the probability of different researchers conducting similar research on the MR-linac, which promotes efficient use, and re-usage of MOMENTUM data.

##### Quality assurance

Data quality is assured by using standardized electronic case report forms (eCRFs) and by the use of automatic validation and verification rules to reduce human error. Also, on-site training of the clinical research coordinators, standard operating procedures (SOPs), and data monitoring will ensure data quality. After one on-site monitoring visit the sites will be monitored through queries and questionnaires for data quality, database completeness, protocol compliance, and compliance to data protection legislation.

#### III. Legal Aspects

##### Legal framework

The MOMENTUM study has been set up as an academic-industrial collaboration managed by the independent not-for-profit organization Lygature. This legal framework was selected because it best reflected the multi-pronged mission of clinical and technical development and clinical assessment. In addition, an academic-industrial partnership was felt to maximize transparency for academic and industrial collaborators.

##### Patient confidentiality

All patients over 18 years old treated on the MR-linac are eligible for participation in the MOMENTUM study. They provide informed consent for the collection of their pseudonymized clinical and technical patient data. Participants can consent to the collection of additional patient reported outcomes and additional MRI scans on the MR-linac. Furthermore, participants from the Netherlands can decide not to share their data with Elekta.

##### Data sharing, data governance, and data access rules

The MOMENTUM study complies with national and regional data processing rules and regulations such as the European General Data Protection Regulation (GDPR), the USA Health Insurance Portability and Accountability Act (HIPAA), and the Canadian Personal Information Protection and Electronic Documents Act (PIPEDA). All rules and regulations on data sharing, data access, and intellectual property are summarized in an Academic Industrial Collaboration agreement (AIC) and signed by the MOMENTUM partners.

As stated in the AIC, every institution that contributed to the MOMENTUM registry will have full control over the patient data they contributed. Therefore, the institutions function as their own data controllers. The UMC Utrecht will monitor registered data of all institutions separately throughout the duration of the study to ensure data quality and adherence to data protection legislation.

Data access is regulated by data access rules which ensure secure, equitable, and safe data handling whilst allowing for open international data exchange in accordance with the FAIR principles. The data access rules are explicitly defined to facilitate research and encourage collaboration between MOMENTUM partners whilst safe-guarding the interests and rights of institutional representatives, TSG representatives, and primary investigators of future research performed on the MR-linac.

For academic partners, we have developed a data request procedure for three different data request types (Table 3). Data requests for each institution's data (type 1 academic research requests) will be granted without further review. Data requests that include patient data from multiple institutions are separated into requests that include patients that are only enrolled in MOMENTUM (type 2 academic research requests) and requests that contain patients that also participate in other studies performed on the MR-linac (type 3 academic research requests). The DMTF will review type 2 and 3 requests for compliance to the institutional protection rule stating that all institutions that contributed significantly to the requested data should be represented by an investigator on the data application form. Otherwise, these institutions must be informed about the data request. Furthermore, these type 2 and 3 requests must also comply with the TSG protection rule, which ensures that these data requests do not compete with ongoing or future research of TSGs. Finally, type 3 academic research requests will require additional approval by the study's coordinating principal investigator (PI protection rule) for data access of patients co-enrolled into both MOMENTUM and other MR-linac studies.

**Table 3:** Data access rules for academic and industry partners within the MOMENTUM study.

**Data access rules for MOMENTUM**		
**Academic research requests**		
**Request**	**Definition**	**DMTF procedure**
Type 1	Institution requests its data	No further review
Type 2	Request for data of multiple institutions	Review request for: - Institutional protection rule - TSG protection rule
Type 3	Type 2 requests that include patients co-enrolled in investigational studies	Conform type 2 request and: - PI protection rule
**Industry research requests**		
**Data class**	**Example**	**DMTF procedure**
Technical patient data	Imaging, RT structure sets	No further review
Classifiers	Patient, disease, and treatment data	Request reviewed for: - Publication intent
Outcomes	Toxicity, cancer control data	Conform classifiers and review of: - Involvement of academic partner
Classifier and Outcome	Combination of the above	Requires unanimous DMTF approval and review of: - Details of data - Endorsement of regulatory submission

*DMTF, Data Management Task Force; RT, radiation therapy*.

*Data protection rules were implemented to ascertain acknowledgment of contributing institutions, Tumor Site Groups (TSG), and principal instigators (PI) in data requests and intended publications*.

Data access by the industry partner Elekta, is governed through separate data access rules. Data requests from the industry partner are categorized into requests for technical patient, classifier, and outcome data (Table 3). Classifier data includes patient, tumor, and treatment characteristics whereas outcome data refers to toxicity and cancer-control related information. Data requests by Elekta require a full description of the requested data elements, cohorts of interest, and the intended use of the data. Requests for technical patient data intended for non-academic purposes are fulfilled without further review by the DMTF. All data requests by Elekta that include classifier and/or outcome data must adhere to the data sharing rules stipulated by the AIC. It is mandatory for the industry partner to have an academic partner involved in data requests relating to outcome data. Data requests from the industry partner that include classifier and/or outcome data require approval by all academic representatives of the DMTF.

### Funding

The MOMENTUM study is financially supported by Elekta AB for 5 years and through in-kind contributions from all participating institutions. Conflicts of interest have been thoroughly addressed by recording academic and industry rights and obligations in the AIC, data access rules, and patient consent forms.

## Discussion

Increased digitalization and technical developments have resulted in the release of promising technical innovations into the medical field. Unlike new pharmacological agents, these innovations and devices undergo limited comparative evaluation as they are approved for release onto the market based on only limited evidence of effectiveness. Acquiring robust data to prove the efficacy and added value of new innovations over standard treatment strategies is challenging and expensive. As a result, there is a lack of high level evidence that supports the use of new technologies in medicine, including in the field of radiation oncology (11).

The MOMENTUM study has been designed to reverse this trend by providing an infrastructure for the evidence-based introduction of MR-linac technology, one of the most recent innovations in radiation oncology (20). Two unique aspects of the MR-linac are the capability of frequent adaptation of the treatment and the integration of functional imaging to facilitate individualized treatments, potentially leading to better disease control and lower toxicity. The MOMENTUM study aims to confirm whether these, for now, theoretical benefits of MR-linac will translate into real benefits for patients. The MOMENTUM study is a unique collaboration between academic and industry partners enabling a systematic approach to the clinical implementation of the Elekta MR-linac without precluding studies that evaluate the MR-linac outside of the MOMENTUM collaboration by providing:

- Industry-funded collaboration with academic institutions aiming to critically evaluate this new technology early in the process of clinical implementation.
- A well-integrated and governed technical and clinical database designed by radiation oncologists.
- Standardized data sets of prospectively accrued data for patients treated with this new technology.
- A methodology framework (R-IDEAL) which TSGs can adopt to demonstrate cancer specific applicability of the MR-linac treatments and ultimately compare MR-linac treatment to standard radiotherapy treatments or alternate treatment approaches.
- Easy and safe international data sharing between all partners, enhancing possibilities for collaborative studies.
- One of only a few industry-funded collaborations aiming to critically analyze the device right at the start of clinical implementation.
- A large scale academic-industrial partnership with all intellectual property rights and data sharing regulations recorded in a collaboration agreement (AIC). Therefore, facilitating industry sponsorship while maintaining an objective environment for academic partners to publish results and to enable Elekta to use pseudonymized technical patient data for further development of the MR-linac.

## Conclusion

The multi-institutional MOMENTUM study was set up to facilitate the evidenced-based implementation of the Elekta MR-linac technology and to support its further technical development. The aim of this new technology is to improve tumor control, survival, and quality of life of cancer patients treated with radiation therapy. The registry study was set up to facilitate the use of the R-IDEAL framework and evaluate the benefit of this technology. This study will facilitate high quality research in the field of radiation oncology.

## Ethics Statement

The study is approved by the Medical Ethics Committee (METC) Utrecht and the local Institutional Review Boards (IRB) of the participating institutions. The patients/participants provided their written informed consent to participate in this study.

## Author Contributions

HV and WH contributed equally to this article. HV, WH, CF, and JC designed the study and are responsible for the general supervision of the study. SO, DE, EB, and HA have been involved in designing the study. SO drafted the manuscript and all authors critically revised the paper. All authors read, provided feedback, and approved the final manuscript.

## Conflict of Interest

JC is Senior Vice President of Medical Affairs and Clinical Research Linac Based RT at Elekta. HA is senior Research Software Engineer at Elekta. KB is Distinguished Scientist at Elekta. AC receives research funding from Elekta. DE is Program Director, Clinical Registries at Elekta. BE receives institutional research and travel support from Elekta. CF-F receives research funding from Elekta. CF received funding and salary support related to the MR-Linac project from the National Institutes of Health (NIH), including: the National Institute for Dental and Craniofacial Research (NIDCR) Academic Industrial Partnership Grant (R01DE028290); NCI Early Phase Clinical Trials in Imaging and Image-Guided Interventions Program (1R01CA218148); an NIH/NCI Cancer Center Support Grant (CCSG) Pilot Research Program Award from the UT MD Anderson CCSG Radiation Oncology and Cancer Imaging Program (P30CA016672) and an NIH/NCI Head and Neck Specialized Programs of Research Excellence (SPORE) Developmental Research Program Award (P50 CA097007). CF received funding and salary support unrelated to this project from: the NIDCR Establishing Outcome Measures Award (1R01DE025248/R56DE025248); a National Institute of Biomedical Imaging and Bioengineering (NIBIB) Research Education Programs for Residents and Clinical Fellows Grant (R25EB025787-01); the NIH Big Data to Knowledge (BD2K) Program of the National Cancer Institute (NCI) Early Stage Development of Technologies in Biomedical Computing, Informatics, and Big Data Science Award (1R01CA214825); National Science Foundation (NSF), Division of Mathematical Sciences, Joint NIH/NSF Initiative on Quantitative Approaches to Biomedical Big Data (QuBBD) Grant (NSF 1557679); NSF Division of Civil, Mechanical, and Manufacturing Innovation (CMMI) standard grant (NSF 1933369). CF receives infrastructure support provided by the Multidisciplinary Stiefel Oropharyngeal Research Fund of the Charles and Daneen Stiefel Center for Head and Neck Cancer and the MD Anderson Program in Image-guided Cancer Therapy. CF has received direct industry grant support, honoraria, and travel funding from Elekta AB. JG was Senior Vice President Medical Affairs at Elekta at time of writing. SH reports non-financial support from Elekta (Elekta AB, Stockholm, Sweden), non-financial support from Merck Sharp and Dohme (MSD), personal fees, and non-financial support from Roche outside the submitted work. EH reports support from Cancer Research UK Programme Grant (A25351) and the National Institute for Health Research (NIHR) Biomedical Research Center at The Royal Marsden NHS Foundation Trust and the Institute of Cancer Research, London. The views expressed are those of the author(s) and not necessarily those of the NIHR or the Department of Health and Social Care. EH reports grants and non-financial support to host institution from Merck Sharp and Dohme, Astra Zeneca, and Bayer, and grants to host institution from Accuray, Varian, Janssen-Cilag, Kyowa Hakko UK, Alliance Pharma (previously Cambridge Laboratories), and Aventis Pharma Limited (Sanofi), outside the submitted work. KH reports support from Elekta MR-Linac Consortium, CRUK ART-NET Network Accelerator Award, CRUK Head, and Neck Programme Grant (C7224/A23275), ICR CRUK Center Grant, ICR/Imperial CRUK Convergence Center Grant and the Royal Marsden Hospital/ICR NIHR Biomedical Research Center. UH receives Elekta and Philips research funding. RH receives institutional funding from Elekta and institutional funding from MSD and ROCHE. AK reports support from Elekta MR-Linac Consortium, CRUK Programme Grant to Royal Marsden/Institute of Cancer Research (ICR) Radiotherapy Physics Department (A19727), and the Royal Marsden Hospital/ICR NIHR Biomedical Research Center. SL receives research funding, honoraria, and travel expenses from Elekta. MN receives research funding from Elekta. KO is program manager of the MOMENTUM project funded by Elekta AB, Stockholm, Sweden. AS is advisor/consultant with Abbvie, Merck, Roche, Varian (Medical Advisory Group), Elekta (Gamma Knife Icon), BrainLAB, and VieCure (Medical Advisory Board). Board Member: International Stereotactic Radiosurgery Society (ISRS). Past educational seminars with Elekta AB, Accuray Inc., Varian (CNS Teaching Faculty), BrainLAB, Medtronic Kyphon. Research grant with Elekta AB Travel accommodations/expenses by Elekta, Varian, BrainLAB. AS also belongs to the Elekta MR Linac Research Consortium, Elekta Spine, Oligometastases and Linac Based SRS Consortia. CS receives Institutional research support from Accuray, Siemens Healthineers, Elekta AB, Philips Healthcare, Manteia Technology and Honoraria and Travel funding from Elekta AB, Accuray. RT receives institutional research support from Elekta and Philips Honoraria (follow ms) and research grants from the Dutch Cancer Society, ZonMw, Health Holland, and NOW. AT received research funding and honoraria from Elekta and research funding from Varian and Accuray. WH receives institutional research and travel support from Elekta. HV receives research funding from Elekta. The remaining authors declare that the research was conducted in the absence of any commercial or financial relationships that could be construed as a potential conflict of interest. The handling editor declared a past co-authorship with one of the authors CS.
